# Case Report: Successful use of emapalumab in adult B-cell acute lymphoblastic leukemia experiencing severe neurotoxicity and hemophagocytic lymphohistiocytosis-like features after CAR-T cell therapy

**DOI:** 10.3389/fimmu.2025.1563736

**Published:** 2025-04-04

**Authors:** Beatrice Manghisi, Giulia Cotilli, Marilena Fedele, Paola Perfetti, Elisabetta Terruzzi, Luisa Verga, Lorenza Maria Borin, Andrea Carrer, Monica Fumagalli, Maria Beatrice Ferrari, Alex Moretti, Roberto Rona, Annalisa Benini, Beatrice Vergnano, Giovanni Palumbo, Alessandra Zincone, Oscar Maglia, Chiara Scollo, Carolina Steidl, Lorenzo Iovino, Adriana Balduzzi, Rocco Piazza, Carlo Gambacorti-Passerini, Matteo Parma, Andrea Aroldi

**Affiliations:** ^1^ Hematology Division, Fondazione IRCCS San Gerardo dei Tintori, Monza, Italy; ^2^ School of Medicine and Surgery, University of Milano-Bicocca, Monza, Italy; ^3^ Pediatric Stem Cell Transplant Unit, Fondazione IRCCS San Gerardo dei Tintori, Monza, Italy; ^4^ Department of Emergency and Intensive Care, Fondazione IRCCS San Gerardo dei Tintori, Monza, Italy; ^5^ Neuroradiology Department, Fondazione IRCCS San Gerardo dei Tintori, Monza, Italy; ^6^ Department of Neurology, Fondazione IRCCS San Gerardo dei Tintori, Monza, Italy; ^7^ Tettamanti Center, Fondazione IRCCS San Gerardo dei Tintori, Monza, Italy; ^8^ Transfusion Medicine Unit, Fondazione IRCCS San Gerardo dei Tintori, Monza, Italy; ^9^ Lymphoma Unit, Department of Onco-Hematology, IRCCS San Raffaele Scientific Institute, Milan, Italy; ^10^ Clinical Research Division, Fred Hutchinson Cancer Center, Seattle, WA, United States

**Keywords:** CAR-T cell therapy, CRS, ICANS, HLH, IEC-HS, emapalumab, leukemia

## Abstract

Chimeric antigen receptor (CAR)-T cell therapy is a powerful adoptive immunotherapy associated with significant toxicity, including cytokine release syndrome (CRS) and immune effector cell-associated neurotoxicity syndrome (ICANS). As CAR-T usage expands, hyperinflammatory toxicities resembling hemophagocytic lymphohistiocytosis (HLH) syndrome are increasingly recognized. Immune effector cell-associated HLH-like syndrome (IEC-HS) describes HLH-like symptoms attributable to CAR-T cell therapy, often presenting as CRS resolves. Treatments for IEC-HS are adapted from primary HLH, including corticosteroids, the recombinant human interleukin (IL)-1 receptor antagonist anakinra and the Janus Kinase inhibitor ruxolitinib. Emapalumab, an anti-IFN-γ antibody, is promising but underexplored in adult IEC-HS cases. We report an adult B-cell acute lymphoblastic leukemia (B-ALL) patient treated with brexucabtagene autoleucel (brexu-cel). The patient developed CRS, refractory neurotoxicity, and IEC-HS with worsening multiorgan failure and hyperinflammatory markers. Treatment included tocilizumab, high-dose corticosteroids, anakinra, siltuximab, and ruxolitinib. Despite aggressive management, hyperinflammation and neurotoxicity persisted. Emapalumab was initiated on day +11, resulting in normalization of the biochemical parameters and full neurological recovery by day +21. The patient recovered from IEC-HS and underwent allogeneic stem cell transplantation. This case highlights the role of emapalumab in managing refractory IEC-HS and persistent neurotoxicity in adults, underscoring the need for targeted interventions in severe CAR-T complications.

## Introduction

Chimeric Antigen Receptor (CAR)-T cell therapy is a well-established immunotherapy known for its efficacy, but it is also associated with significant toxicity, including hyperinflammatory conditions such as Cytokine Release Syndrome (CRS) and Immune effector Cell-Associated Neurotoxicity Syndrome (ICANS) (1). As the use of CAR-T cells continues to expand, there is growing recognition of additional hyperinflammatory toxicities resembling Hemophagocytic Lymphohistiocytosis (HLH) syndrome (2). The term Immune Effector Cell-associated HLH-like Syndrome (IEC-HS) has been introduced to describe HLH-like symptoms attributable to IEC therapy. These symptoms typically present with a delayed onset, when CRS is about to resolve (2). Suggested treatment strategies for IEC-HS are adapted from those used to manage primary HLH, adopting a sequential approach, particularly in refractory cases. This includes the use of corticosteroids, the recombinant human interleukin (IL)-1 receptor antagonist anakinra and the Janus Kinase inhibitor ruxolitinib (2). Emapalumab, an anti-interferon-γ (IFN-γ) antibody, is approved for the treatment of primary HLH, but its use for the treatment of IEC-HS following CAR-T cell therapy, especially in adults, is limited and requires further investigation (3–6).

Here we report the case of an adult patient with B-cell acute lymphoblastic leukemia (B-ALL) treated with brexucabtagene autoleucel (brexu-cel), who developed CRS, refractory neurotoxicity and IEC-HS. Given the aggressiveness of the IEC-HS and the inefficacy of other therapeutic options, we hypothesized that emapalumab could effectively address refractory IEC-HS and neurotoxicity following CAR-T cell therapy in the setting of adult B-ALL.

## Case report

A patient in their 30s presented with leukocytosis (white blood cells (WBC): 22.00 x 10^9^/L) and circulating blasts. Bone marrow aspirate (BMA) revealed 90% of atypical lymphoblasts and immunophenotyping was consistent with B-ALL, common phenotype (positivity for CD19, CD10, CD22). Cytogenetics revealed normal karyotype and next-generation sequencing analysis was negative for additional mutations. Staging evaluations showed no extramedullary involvement.

The patient started chemotherapy according to the GIMEMA LAL1913 protocol (7), achieving morphological complete remission (CR) with positivity of minimal residual disease (MRD: 5 × 10^-4^) post-cycle C1 (+1 month from diagnosis). Cycles C2 and C3 were administered but MRD progression post-cycle C3 was documented (MRD: 4.9 × 10^-^², +3 months), which required the start of second-line therapy with blinatumomab, achieving morphological CR with MRD reduction (MRD: 5 x 10^-4^; +4 months). A second cycle of blinatumomab was performed but at the end of this regimen relapse occurred (+6 months). BMA identified 10% of lymphoblasts, whose phenotype showed two different blast subpopulations, one negative for CD19 surface expression. Since the patient was not a candidate for CAR-T cells at the time, CD22 antigen was homogeneously expressed and third-line treatment with inotuzumab ozogamicin (IO; +7 months) was started. Despite achieving a morphological CR after two cycles of IO, MRD positivity was still present (2 x 10^-3^ after IO – cycle 2) and relapse eventually occurred with hyperleukocytosis (+8 months; WBC: 122.00 x 10^9^/L). In this case, lymphoblasts immunophenotyping broadly expressed CD19, suggesting that CD19 negativity, previously documented in one blast subpopulation, was secondary to antigen downregulation that resolved after blinatumomab hold (8).

**Figure 1:**
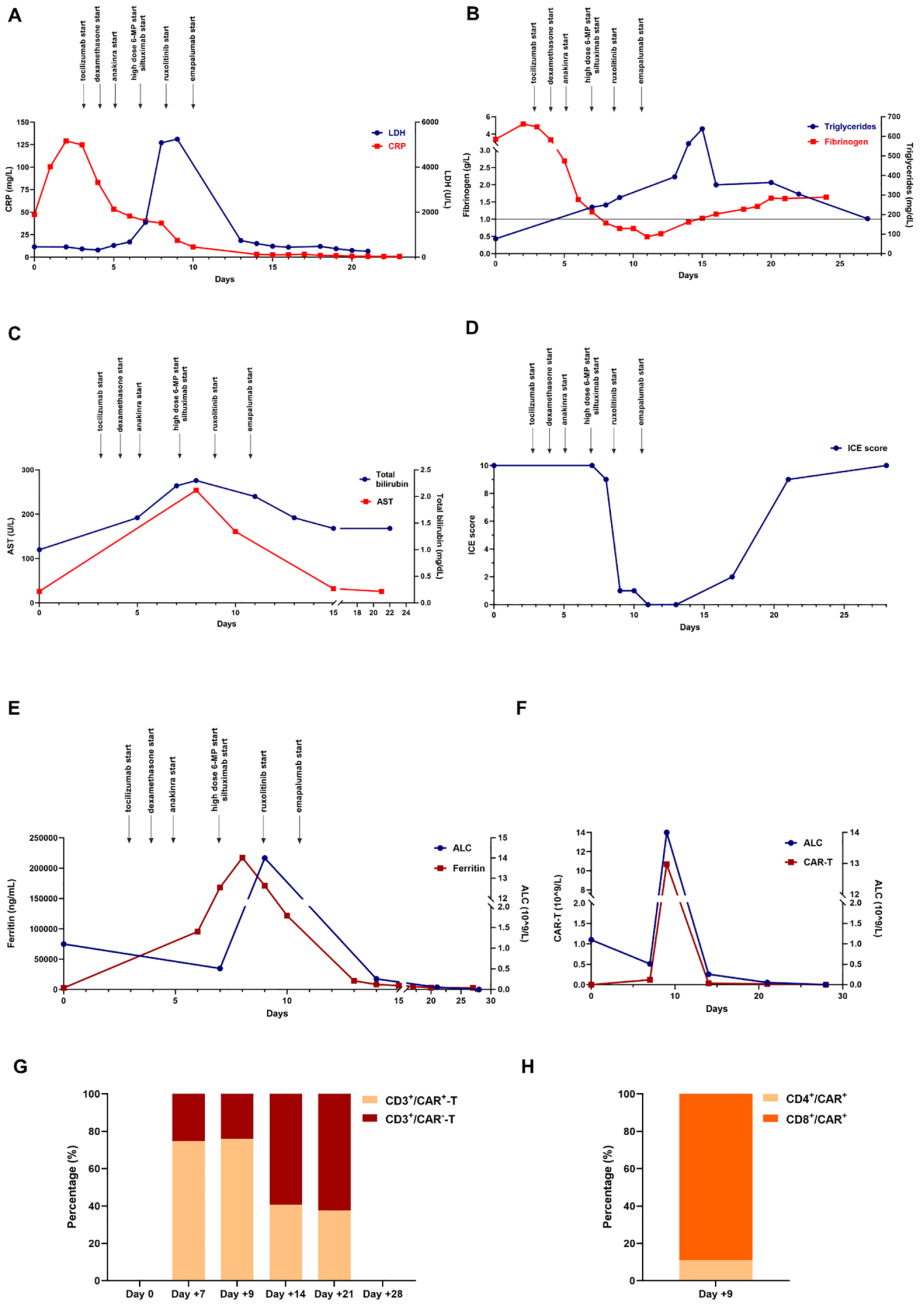
**(A-E)** LDH, CRP, fibrinogen, triglycerides, liver function tests, ICE score, ferritin, ALC monitoring and treatment interventions over time after CAR-T cell infusion. **(F)** Expansion of CAR-T cells reflects the occurrence of lymphocytosis on day +9 after infusion. **(G)** Percentage of CAR-T population over time with respect to total CD3+ T cell population. **(H)** CD4/CD8 ratio in CAR-T population on day +9 post-infusion. LDH, lactate dehydrogenase; CRP, C-reactive Protein; ICE score, immune effector cell encephalopathy score; AST, aminotransferase; CAR-T cell, chimeric antigen receptor-T cell therapy; ALC, absolute lymphocyte count; 6-MP, 6-methylprednisolone.

Since hyperleukocytosis and high circulating blasts can compromise leukapheresis and CAR-T manufacturing, debulking therapy with steroids and cyclophosphamide was conducted to reduce WBC to a value inferior to 40.00 x 10^9^/L, as suggested in the pediatric setting (9). Lymphocyte apheresis was performed, collecting 2 x 10^9^ CD3^+^ T cells, and shipped for brexu-cel manufacturing. Bridging therapy with fludarabine and high-dose cytarabine was provided while awaiting CAR-T production. After 4 weeks of iatrogenic aplasia, the patient experienced disease recurrence immediately after the start of the lymphodepleting regimen (cyclophosphamide and fludarabine). Levetiracetam prophylaxis (750 mg/bid, oral administration) was introduced at the initiation of lymphodepletion and brexu-cel was finally infused (40 days after lymphocytes collection). On day +2 post-infusion, the patient experienced grade 1 CRS (fever) which only required symptomatic treatment (acetaminophen). On day +3, CRS turned into grade 2 (persistent fever, hypotension partially responsive to fluid hydration, oxygen support with low-flow nasal cannula), with increase in C-reactive Protein (CRP) levels (**Figure 1A**), and tocilizumab was started (8 mg/kg/tid intravenous (IV), for 24 hours). On day +4, refractoriness to tocilizumab and the need for increased oxygen support (Venturi mask 50%), persistent fever, hypotension, low urine output and diffuse subcutaneous edema, mimicking capillary leak syndrome, were documented. Due to the clinical pattern, consistent with grade 3 CRS, dexamethasone (10 mg IV, every 6 hours) was started. After 24 hours of dexamethasone, as steroid monotherapy had failed to control CRS, anakinra was initiated on day +5 (100 mg IV every 6 hours). On day +7, the patient required intensive care unit (ICU) admission due to worsening clinical conditions, characterized by persistent fever, increase in oxygen support (continuous positive airway pressure – CPAP), trilinear cytopenia, multiorgan failure with impaired kidney function and electrolyte disorder (hyperkalemia, hyperphosphatemia). In addition, CRP levels dropped after tocilizumab treatment, but impaired levels of lactate dehydrogenase (LDH), fibrinogen, liver function tests (LFTs), triglycerides and ferritin raised concern for the onset of HLH-like features (**Figures 1A–E**). Therefore, management of IEC-HS was initiated (2). On day +7, 6-methylprednisolone bolus was started (1000 mg IV daily for three days, then tapered) and anakinra was increased up to the maximum dose level (8 mg/kg/day, 200 mg IV every 6 hours). On day +7, siltuximab was added (dose 11 mg/kg IV) to manage refractory CRS and prevent ICANS, which was likely to occur, based on the CRS presentation (2).

**Figure 2:**
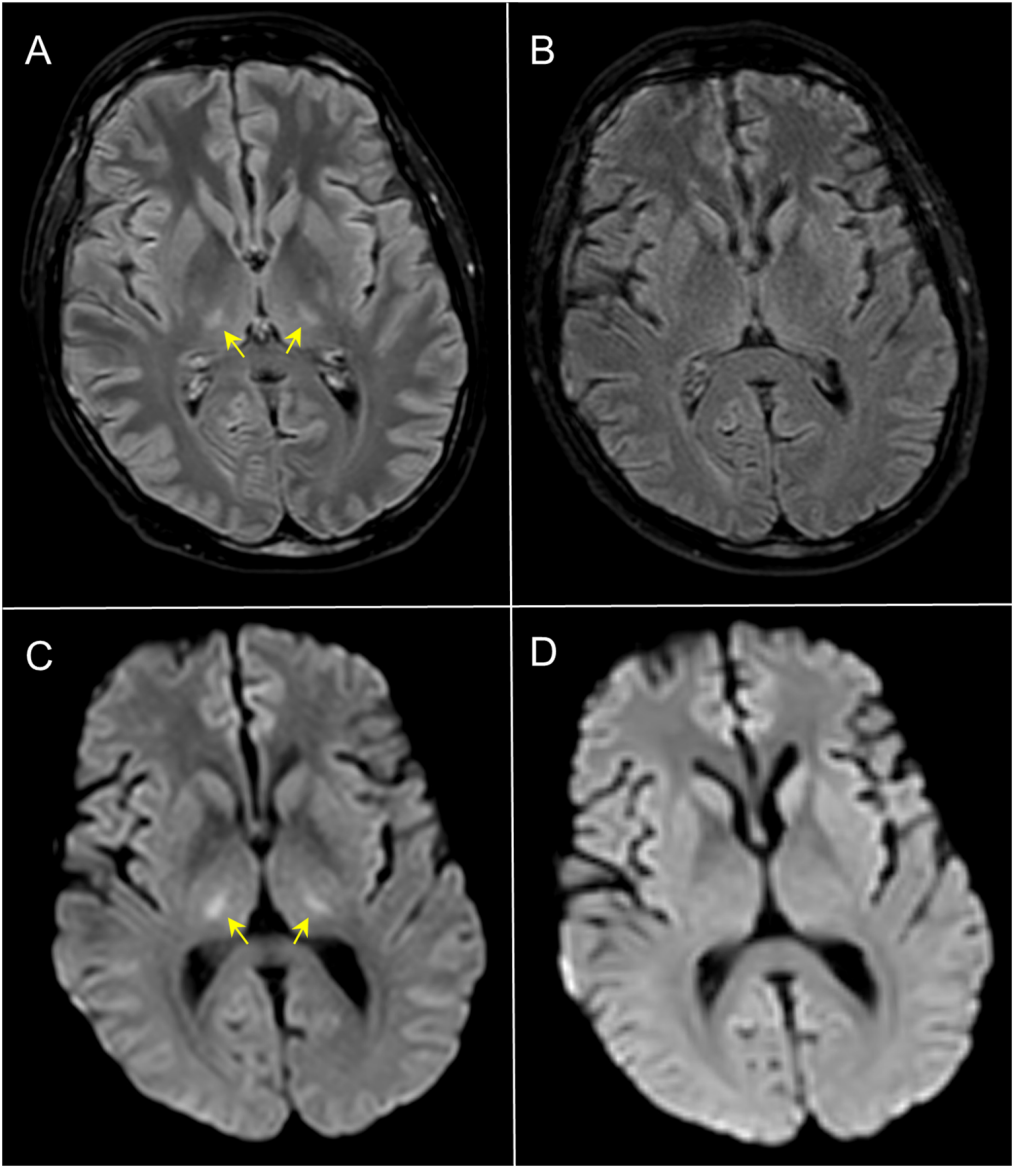
Axial 3D-FLAIR **(A, B)** and DWI **(C, D)** MRI. At day +13, 3D-FLAIR showed symmetrical faint hyperintensity of the thalami with corresponding restricted diffusivity (yellow arrows in **(A, C)** consistent with ICANS; follow-up imaging at day +23 **(B, D)** demonstrated complete normalization of the signal abnormalities.

During ICU admission, the patient required continuous renal replacement therapy (CRRT), CPAP support and negative fluid balance. Meanwhile, biochemical abnormalities were progressively increased, suggesting the development of an overt IEC-HS (ferritin peak on day +8: 217412 ng/mL; reference range: 30-400 ng/mL) (**Figure 1D**). In addition, the patient manifested a dramatic expansion of CAR-T cells, showing a maximum peak on day +9 (10.68 x 10^9^/L), associated with lymphocytosis (**Figures 1D–G**). Abnormal expansion of CAR-T cells could have explained the onset of IEC-HS and, to this end, ruxolitinib was added on day +9 (10 mg/bid, oral administration). Despite the ongoing treatment, on day +9, neurological manifestation started to develop, showing confusion, global aphasia, fluctuating consciousness until coma (grade 4 ICANS). Intubation and deep sedation were conducted to alleviate neurological symptoms, and a second dose of siltuximab was administered on day +9. Electroencephalography showed metabolic encephalopathy pattern without seizure abnormalities, and brain Magnetic Resonance Imaging (MRI) revealed focal symmetrical hyperintensity in the thalami, a known radiological pattern of ICANS (**Figures 2A–C**) (1). The slow decrease in biochemical parameters, persistent elevated ferritin levels, as well as continuous neurological alterations with no improvement after any attempt of emerging from sedation, were conclusive for ongoing hyperinflammation and refractory IEC-HS (**Figures 1A–D**). Compassionate use of emapalumab was approved and IV infusion started from day +11 (recommended dose: 1 mg/kg twice weekly for two weeks) (2). Seventy-two hours after treatment initiation, ferritin level dramatically decreased (from 121756 ng/mL to 8337 ng/mL), LFTs gradually normalized and CRRT was interrupted (**Figures 1A–D**). Neurological symptoms eventually resolved, allowing for the discontinuation of deep sedation on day +18 (seven days after emapalumab started) (**Figure 1C**). The patient completely recovered from IEC-HS without any sequalae and was discharged from the ICU on day +21. Emapalumab was well tolerated and the only side effect was moderate gastrointestinal bleeding (melena due to gastric erosions seen by esophagogastroduodenoscopy), responsive to supportive therapy and drug interruption (cumulative dose infused: 300 mg). The brain MRI follow-up on day +23 showed complete resolution of thalamic abnormalities (**Figures 2B–D**). On day +35, the patient repeated BMA (no lymphoblasts detected; non-diagnostic assessment of MRD) and on day +60 underwent allogeneic stem cell transplantation (alloSCT) to consolidate CR following brexu-cel. Day +30 BMA post-alloSCT showed CR with low-level MRD (5 x 10^-4^) and, on day +60 post-alloSCT, the patient relapsed with CD19-negative B-ALL and passed away for disease progression on day +90.

## Discussion

This case underscores the critical role of immune-mediated complications following CAR-T cell therapy, particularly the progression from CRS to IEC-HS, where macrophages and effector cells predominantly drive pathophysiology through the production of IFN-γ (2). IFN-γ acts as the central driver of the inflammatory loop, initiating a cascade that activates downstream pathways beyond IL-6. This explains the limited efficacy of single-agent therapies, effective only in early stages, and highlights the need for broad-spectrum approaches in advanced phases to suppress multiple cytokines and break the IEC-HS inflammatory loop (1, 2). Some risk factors for IEC-HS overlap with those for CRS, including high disease burden and baseline inflammation, while others, such as the CAR-T cell antigen target (e.g., CD22), may be specific to IEC-HS; however, further research is required to fully elucidate its biology (2). Emapalumab, by targeting IFN-γ produced by both macrophages and CAR-T cells, effectively disrupts the inflammatory loop and allows to achieve therapeutic outcomes not seen with other agents (2–4, 6).

Even though pathophysiology has been thoroughly outlined in recent years and can effectively address therapeutic management, diagnosis of IEC-HS may be difficult and should take into account different clinical and lab parameters. In our case, we did not have the opportunity to measure markers like IFN-γ and soluble IL-2 receptor (sIL-2r), which are known to be involved in the pathogenesis of HLH and IEC-HS, becoming helpful in the diagnostic assessment (2). Despite this limitation, we made a confident diagnosis of IEC-HS based on the delayed onset of impaired multiple markers such as ferritin levels, LDH, AST, fibrinogen and total bilirubin, all of them known to be altered when IEC-HS and HLH occur (2). Moreover, all these markers and the clinical manifestations were associated with high probability of reactive HLH according to the HScore based on the evaluation of the whole clinical picture (i.e., underlined immunosuppression, persistent fever, persistent trilinear cytopenia, elevated AST, ferritin and triglycerides levels; HScore: >99% probability of HLH) (2). Unfortunately, because of dry tap at BMA, it was not possible to assess the presence of hemophagocytosis within bone marrow. Nevertheless, according to IEC-HS and HScore criteria, the diagnosis of HLH does not necessarily require BMA, since the combination of clinical and laboratory findings could set a diagnosis of HLH without the need for bone marrow morphology (2). Even though hemophagocytosis can be a supportive finding, its absence does not rule out HLH in the setting of IEC-HS (2).

Moreover, the possibility of distinguishing between early onset of CRS and late occurrence of HLH was sustained by the worsening of pivotal markers, not altered during CRS phase (i.e., sharp rise in ferritin and AST levels, coagulopathy with low fibrinogen), documented later, in line with the typical timing of IEC-HS manifestation (2). Specifically, ferritin levels spiked rapidly around days 7-8 post-infusion and HLH alterations presented only after CAR-T cell expansion, a pattern that is characteristic of IEC-HS as described in ASTCT recommendations (2).

Although, in our case, some parameters showed an initial decrease before the use of emapalumab, key markers such as fibrinogen and triglycerides specifically improved after its introduction. While AST and ferritin levels began to decline earlier, likely due to multiple therapeutic interventions, a significant drop was found only after emapalumab treatment. In addition, clinical resolution was obtained exclusively following its initiation, suggesting its pivotal role in IEC-HS treatment. Notably, neurological and radiological abnormalities worsened over time and were unresponsive to the intensive treatment provided by expert recommendations (2).

Emapalumab was the only therapy that effectively resolved the neurological impairment and the refractory HLH manifestations. As a matter of fact, emapalumab was crucial in addressing both systemic hyperinflammation in IEC-HS and localized neurotoxicity as, unlike other drugs suggested for IEC-HS like ruxolitinib, it can cross the blood-brain barrier without impairing systemic CAR-T cell activity (3–6, 10).

With the limitations and the difficulties found in the diagnostic assessment, we showed in our case that emapalumab represents a pivotal intervention for severe, refractory IEC-HS, providing a targeted, pathophysiology-driven therapy that supports its broader consideration not only in pediatric but also adult CAR-T cell recipients.

## Data Availability

The datasets presented in this article are not readily available because we do not have raw data to share in this case report. Any other concerns may be turned to the corresponding author upon reasonable request. Requests to access the datasets should be directed to andrea.aroldi@unimib.it.
